# Microwave synthesis of ultrasmall gold nanoparticles, their γ-globulin interaction and subsequent effects in hepatocellular carcinoma cells

**DOI:** 10.1186/s13065-026-01868-0

**Published:** 2026-07-02

**Authors:** Gong Chen, Shanshan Han, Xiaoyan Huang, Weiping Wei

**Affiliations:** 1 Department of General, Visceral & Transplant Surgery, Section Surgical Research, University of Heidelberg, 69120 Heidelberg, Germany; 2 First Department of Medicine, Medical Faculty Mannheim, University Medical Centre Mannheim (UMM), Heidelberg University, 68167 Mannheim, Germany

**Keywords:** Ultrasmall gold nanoparticles, Microwave-assisted synthesis, Protein corona formation, γ-globulin interaction, Hepatocellular Carcinoma Cells

## Abstract

Gold nanoparticles (GNPs) are widely explored for biomedical applications; however, their interactions with plasma proteins and the resulting effects on biological activity remain insufficiently understood. In this study, ultrasmall GNPs were synthesized via a solvent-polarity-controlled microwave-assisted reduction method using sodium citrate as a reducing agent and polyvinylpyrrolidone as a stabilizer. The synthesized GNPs exhibited a mean diameter of 6 ± 2 nm and were subsequently employed to investigate the formation and biological implications of a γ-globulin protein corona. Physicochemical characterization demonstrated that γ-globulin adsorption significantly altered the colloidal properties of GNPs, resulting in increased hydrodynamic size, reduced zeta potential, and a red-shift of the surface plasmon resonance band. Fluorescence quenching studies revealed a spontaneous interaction between GNPs and γ-globulin. Thermodynamic analysis indicated that the binding process was predominantly driven by hydrogen bonding and van der Waals interactions. Molecular docking using Au₁₃ and Au₄₃ clusters as computationally tractable models of the GNP surface further confirmed the involvement of polar amino acid residues in protein adsorption. Spectroscopic analyses showed only minor conformational perturbations in γ-globulin following interaction with GNPs. Biological evaluation demonstrated that bare GNPs exerted significant selective antiproliferative activity against HepG2 hepatocellular carcinoma cells relative to normal human hepatic L-02 cells, whereas γ-globulin-coated GNPs exhibited markedly reduced cytotoxicity. Protein corona formation decreased cellular uptake, attenuated reactive oxygen species generation, preserved antioxidant defenses, and reduced apoptosis-related signaling. These findings establish a direct mechanistic relationship between γ-globulin corona formation, nanoparticle physicochemical properties, cellular uptake, and biological response, highlighting the critical role of the nano–bio interface in determining the therapeutic performance of GNP-based systems.

## Introduction

Gold nanoparticles (GNPs) have emerged as versatile nanomaterials in numerous bioscience and biomedical applications, including cancer therapy, targeted drug delivery, biosensing, and catalysis [1]. Their broad applicability primarily arises from their distinctive surface plasmon resonance (SPR) properties, which confer unique optical and physicochemical characteristics at the nanoscale. Notably, GNPs have demonstrated promising anticancer activity against various malignancies, including lung, ovarian, breast, prostate, and cervical cancers [2]. Given the rapidly increasing demand for GNPs in therapeutic and diagnostic contexts, considerable research efforts are directed toward the synthesis of monodisperse NPs with controlled physicochemical properties and toward understanding their interactions with biological macromolecules.

The physicochemical characteristics of GNPs—such as size, morphology, surface charge, and colloidal stability—can be tailored through careful control of synthesis parameters, including the choice of reducing and stabilizing agents, reaction temperature, pH, precursor concentration, and solvent system. Among the various approaches developed for GNP synthesis, including biological [3, 4], chemical [5, 6], and physical [6] methods, microwave-assisted chemical synthesis has emerged as an attractive strategy owing to its rapid volumetric heating, uniform energy distribution, and enhanced nucleation kinetics [7]. However, prolonged exposure to high microwave irradiation power can induce NP aggregation or structural degradation as a consequence of localized superheating [8]. One effective strategy to mitigate these limitations involves coupling microwave-assisted synthesis with modulation of the solvent polarity. For instance, Hussain et al. demonstrated that decreasing solvent polarity significantly contributes to the formation of ultrasmall and monodisperse GNPs [9]. Accordingly, ethanol–water mixtures provide an advantageous reaction medium, enabling controlled nucleation and growth processes during microwave synthesis of GNPs.

Protein-based templates, including albumin, antibodies, and various cytokines, have been employed to synthesize biocompatible nanocomposites for drug delivery applications [10]. Therefore, understanding the nano–bio interface, particularly the interaction of NPs with plasma proteins and the consequent formation of a protein corona, is critical for predicting NP biocompatibility, biodistribution, pharmacokinetics, and pharmacodynamics. In human plasma, albumin, γ-globulin, and fibrinogen represent the major protein fractions, accounting for approximately 54%, 38%, and 7% of the total protein content, respectively [11]. The adsorption of these proteins onto the NP surface can significantly alter the NPs’ biological identity. For example, GNPs with spherical morphology generally induce less structural perturbation in proteins compared with rod-shaped NPs, although surface functionalization—such as PEGylation—can markedly influence the degree of protein denaturation [12]. Spectroscopic studies have further demonstrated that the interaction of GNPs with serum proteins typically exhibits moderate binding affinity, accompanied by minimal conformational alterations in the protein secondary structure.

Among plasma proteins, γ-globulin—characterized predominantly by β-sheet secondary structure—can bind a broad spectrum of small molecules, drugs, and nanomaterials. Previous studies have shown that γ-globulin interacts strongly with chemically synthesized GNPs (~ 10 nm), exhibiting a binding constant of approximately K_b_ = 10⁹ L mol⁻¹, with a single dominant binding site that can induce notable conformational changes in the protein structure [13]. Nevertheless, excessively strong NP–protein binding may adversely affect the biological performance of nanomaterials. Tight adsorption may reduce NP aggregation but can simultaneously attenuate their biological activity and cellular interactions. Conversely, weaker interactions may influence pharmacokinetic behavior, circulation time, and tissue infiltration. These challenges can potentially be addressed by modulating the physicochemical properties of NPs, thereby tuning their binding affinity toward plasma proteins.

Hepatocellular carcinoma (HCC) represents one of the most prevalent and lethal malignancies worldwide, exhibiting a high global incidence and mortality rate. Because the underlying etiology of HCC remains incompletely understood, effective primary prevention strategies are still under investigation. Consequently, secondary prevention—emphasizing early detection, accurate diagnosis, and timely therapeutic intervention—remains critically important. Although GNPs have been widely explored as potential anticancer nanotherapeutics, the precise molecular mechanisms through which these NPs, particularly in the presence of protein corona complexes, exert growth-inhibitory effects on cancer cells remain largely unclear.

In the present study, GNPs were synthesized using a microwave-assisted reduction method (90 W, 5 min) in a solvent system containing 20% ethanol to reduce medium polarity. Sodium citrate was employed as the reducing agent, while polyvinylpyrrolidone (PVP) served as a stabilizing and capping agent to enhance colloidal stability. Following physicochemical characterization of the synthesized GNPs, their interaction with γ-globulin was systematically investigated using various spectroscopic and theoretical approaches to elucidate the NP–protein binding mechanism. Subsequently, the anticancer activity of bare GNPs and GNP–γ-globulin complexes was evaluated against hepatocellular carcinoma cells using a series of cellular and molecular assays.

## Materials and methods

### Materials

γ-Globulin (∼99%, lyophilized powder), 3-(4,5-dimethylthiazol-2-yl)-2,5-diphenyl-2 H-tetrazolium bromide (MTT), HAuCl_4_∙_3_H2O (≥ 99%), sodium citrate (Na_3_Ct), polyvinylpyrrolidone (PVP) K60, and ethanol (99.90%) were acquired from Sigma-Aldrich, USA. To perform the biochemical assays, the ELISA-relevant kits were purchased from ThermoFisher Scientific, USA. All aqueous solutions were prepared using double-distilled water (ddH₂O). γ-Globulin was dissolved in 10 mM phosphate buffer (pH 7.40, measured at 25 °C). All reagents were of analytical grade and used without further purification.

### Synthesis of gold nanoparticles (GNPs) and determination of their concentration

To reduce solvent polarity, Na3Ct was dissolved in an ethanol–water mixture containing 20% (v/v) ethanol. PVP was subsequently added to a final concentration of 1% (w/v), and the mixture was sonicated for 2 h. The pH of the solution was adjusted to 10.5 using 0.1 M NaOH and monitored with a calibrated pH meter. HAuCl4 aqueous solution was then added, and the final reaction volume was adjusted to 10 mL. The final concentrations of HAuCl4 and Na3Ct in the reaction mixture were 0.88 mM and 10 mM, respectively. The reaction mixture was exposed to microwave irradiation (90 W in a household microwave oven) for 5 min. The concentration of GNPs was calculated by UV–vis absorbance according to the reported extinction coefficient for GNPs [14].

### Protein corona formation

To prepare the protein corona, γ-globulin was incubated with ultrasmall GNPs at a fixed molar ratio of 1:1. The incubation was carried out in 10 mM phosphate buffer (pH 7.40, measured at 25 °C) for 1 h at room temperature under gentle orbital shaking to facilitate uniform adsorption of γ-globulin onto the GNP surface. After incubation, GNP–γ-globulin complex was separated from excess unbound γ-globulin by centrifugation at 12,000 × g for 30 min at 4 °C. The supernatant containing unbound γ-globulin was carefully removed, and the pellet was resuspended in fresh phosphate buffer.

### Characterization of gold nanoparticles (GNPs) and GNPs–γ-globulin complex

X-ray diffraction (XRD) analysis of GNP was recorded on a Rigaku diffractometer (Cu-Kα radiation, λ = 0.1546 nm) at 40 kV and 40 mA in the 2θ range 30–80°. Microscopy analysis of GNPs and GNPs–γ-globulin was carried out using a JEOL JEM-2010 transmission electron microscopy (TEM) at a voltage of 100 kV. Size distribution and zeta potential of synthesized GNPs and GNPs–γ-globulin were determined using the dynamic light scattering (DLS) method on a Zetasizer Nano ZS (ZEN3600 and Malvern, UK). UV–visible spectra of synthesized GNPs and GNPs–γ-globulin were recorded using a Hitachi spectrophotometer (Japan) from 400 to 800 nm wavelength at ambient temperature. For the analysis of the size, zeta potential and SPR properties of GNPs in the presence of γ-globulin, the molar ratio of protein: GNP was fixed to 1:1.

### Fluorescence spectroscopy measurements

The fluorescence spectra of γ-globulin either alone or with different concentrations of GNPs were read in the wavelength range of 300–420 nm following excitation at 280 nm on a Hitachi F-4500 spectrofluorophotometer (Hitachi Company, Japan). The concentrations of γ-globulin were fixed at 5 µM and the concentrations of GNPs were in the range of 0.1–2 µM. The slit widths for excitation and emission were set at 5 nm. The fluorescence intensities were corrected against absorption and reabsorption to exclude the inner filter effect using the equation reported previously [13]. Also, the fluorescence intensity of protein was corrected against GNP fluorescence intensity.

### Synchronous fluorescence spectroscopy measurements

Synchronous fluorescence spectra were detected following scanning γ-globulin at Δλ of 15 nm (Tyr residues) and 60 nm (Trp residues), respectively. All other experimental procedures remained identical to Sect.  2.6.

### Circular dichroism (CD) measurements

The CD spectra of γ-globulin either alone or with different concentrations of GNPs were recorded on a Jasco spectropolarimeter (J-810–150 S) equipped with a 0.1 cm path length cuvette. Scan speed and response time were fixed at 500 nm min^− 1^ and 0.5 s, respectively. Each spectrum was corrected against the buffer solution and appropriate baseline correction was done. The concentration of γ-globulin was kept at 10 µM and different molar ratios of GNPs (0.2–4 µM) were added to the protein solution.

### Molecular docking analysis

A molecular docking study was performed to investigate the docking of γ-globulin and GNP surface using AutoDock software. Nonocrystal webtool was used to construct gold spherical clusters with two sizes of 13 and 43 atoms [15]. These Au clusters were used as a computationally tractable model of the GNP surface to identify potential IgG binding sites. The γ-globulin structure with ID 1AJ7 was downloaded from the Protein Data Bank, while hydrogen atoms were added, charges were assigned, and water molecules and other ligands in the crystal structure were removed by AutoDock Tools. The GNP surfaces were also optimized by adding hydrogens and assigning charges. The docking box was set to 126 × 126 × 126x Å with a spacing of 1 Å. The docking outcomes were analyzed by AutoDock Tools, while the binding energy and different parameters and forces were analyzed using Discovery Studio.

### Cell culture

Hepatocellular carcinoma (HCC) HepG2 cells and normal human hepatic L-02 cells were purchased from the Cell Bank of the Type Culture Collection of the Chinese Academy of Sciences (Shanghai, China). The cells were cultured in Dulbecco’s modified Eagle’s medium (DMEM; Gibco, Carlsbad, CA, USA) supplemented with 10% (v/v) fetal bovine serum (FBS; Gibco), 100 U/mL penicillin, and 100 µg/mL streptomycin. Cells were maintained at 37 °C in a humidified incubator containing 5% CO₂.

### MTT assay

The impact of GNPs at different concentrations (0.1–50 µM) either alone or in the presence of γ-globulin (with a molar ratio of 1:1) on the proliferation of cells was assessed by MTT assay. The cells seeded on the 96-well plate and grown for 24 h were exposed to increasing concentrations of GNP or GNPs–γ-globulin complex and further incubated for 24 h. Then, 20 µl MTT was added to each well, incubated for 4 h, and mixed with 100 µl DMSO. Finally, the optical density of samples was read on a microplate reader at 570 nm.

The half-maximal inhibitory concentration (IC₅₀) values of GNPs and GNPs–γ-globulin complexes against HepG2 cells were calculated from MTT assay data using nonlinear regression analysis. Cell viability (%) was plotted against the logarithm of NP concentration (0.1–50 µM), and the data were fitted to a four-parameter logistic (4PL) sigmoidal dose–response model (Hill equation) [16].

1 $$Y=\mathrm{Min}+\frac{\mathrm{Max}-\mathrm{Min}}{1+\left(\frac{X}{IC_{50}}\right)}$$

where *Y* represents the percentage of viable cells, *X* is the concentration of the tested sample, *Min* and *Max* correspond to the minimum and maximum responses, respectively, and *H* is the Hill slope coefficient. The IC₅₀ value was defined as the concentration of GNPs required to inhibit 50% of cell viability relative to untreated control cells.

### Intracellular GNP detection

After treatment with IC_50_ concentration of bare GNPs or GNPs- γ-globulin (with a molar ratio of 1:1) for 24 h, the cells were used for intracellular Au content measurements using an inductively coupled plasma optical emission spectroscopy (ICP, iCAP 6300 of Thermo scientific) as previously stated [17].

### Intracellular ROS detection

Intracellular ROS production was assessed by a ROS assay kit (ThermoFisher Scientific, USA). After treatment with IC_50_ concentration of GNPs for 24 h, the HCC HepG2 cells were incubated with 10 µM DCFH-DA for 30 min at 37 °C and washed with PBS three times. Intracellular ROS was then determined by detecting the fluorescence intensity using a fluorimeter at an excitation wavelength of 488 nm and an emission wavelength of 525 nm [18].

### Quantification of oxidative stress biomarker levels

The control and GNP-treated HCC HepG2 cells for 24 h were collected and lysed using lysis buffer. Then the suspension was centrifuged at 4 °C and the protein concentration in the collected supernatants was measured and diluted in assay buffer. The glutathione (GSH) level, SOD activity, and CAT activity were assessed by respective ELISA assay kits following the recommended protocols of the kit’s manufacturer (ThermoFisher Scientific, USA).

### Measurement of protein expressions by ELISA

The expression levels of Bcl-2, Bax, cytochrome c, and caspase-3 in the cell lysates of the control and GNP-treated HCC HepG2 cells were assessed using the commercial assay kits based on the protocols provided by the manufacturer (ThermoFisher Scientific, USA). Briefly, after treatments and homogenization of the cells in the lysis buffer, the cell lysates were vortexed and centrifuged followed by the collection of supernatants. Then, protein concentration was determined in the supernatant using the BCA kit (Sigma, USA) and the protein expression levels of caspase-3, cytochrome c, Bax, and Bcl-2 were done by ELISA assay.

### Measurement of mRNA expressions by qRT-PCR

The control and GNP-treated HCC HepG2 cells for 24 h were collected and total RNA was extracted using Trizol reagent (Takara, Dalian, China) in accordance with the manufacturer’s protocols, followed by determination of RNA purity and concentration by spectrophotometry. RNA was reversely transcripted to cDNA using the cDNA Reverse Transciption Kit (Takara, Dalian, China) in accordance with the manufacturer’s instruction. PCR was carried out based on the standard protocols using a Real-Time PCR-system (BioSystems) with SYBR Green master mix (Applied Biosystems, Warrington, United Kingdom).

### Statistical analysis

Results were analyzed by one-way analysis of variance (ANOVA) followed by Dunnett’s test or Turkey’s test. The data was presented as mean ± standard deviation (SD). The value of *p* < 0.05 was considered as statistically significant.

## Results and discussion

This section presents a systematic investigation of the interaction between γ-globulin and ultrasmall GNPs, focusing on the formation and stability of the protein corona and its impact on the physicochemical and biological properties of the NPs. Key analyses include changes in SPR, hydrodynamic size, and zeta potential, complemented by spectroscopic and theoretical studies to elucidate the binding mechanism. Functional assays further reveal how protein corona formation modulates cellular effects, providing insight into the interplay between GNP surface chemistry (protein corona) and biological response.

### Characterization of synthesized ultrasmall gold nanoparticles (GNPs)

**Fig. 1:**
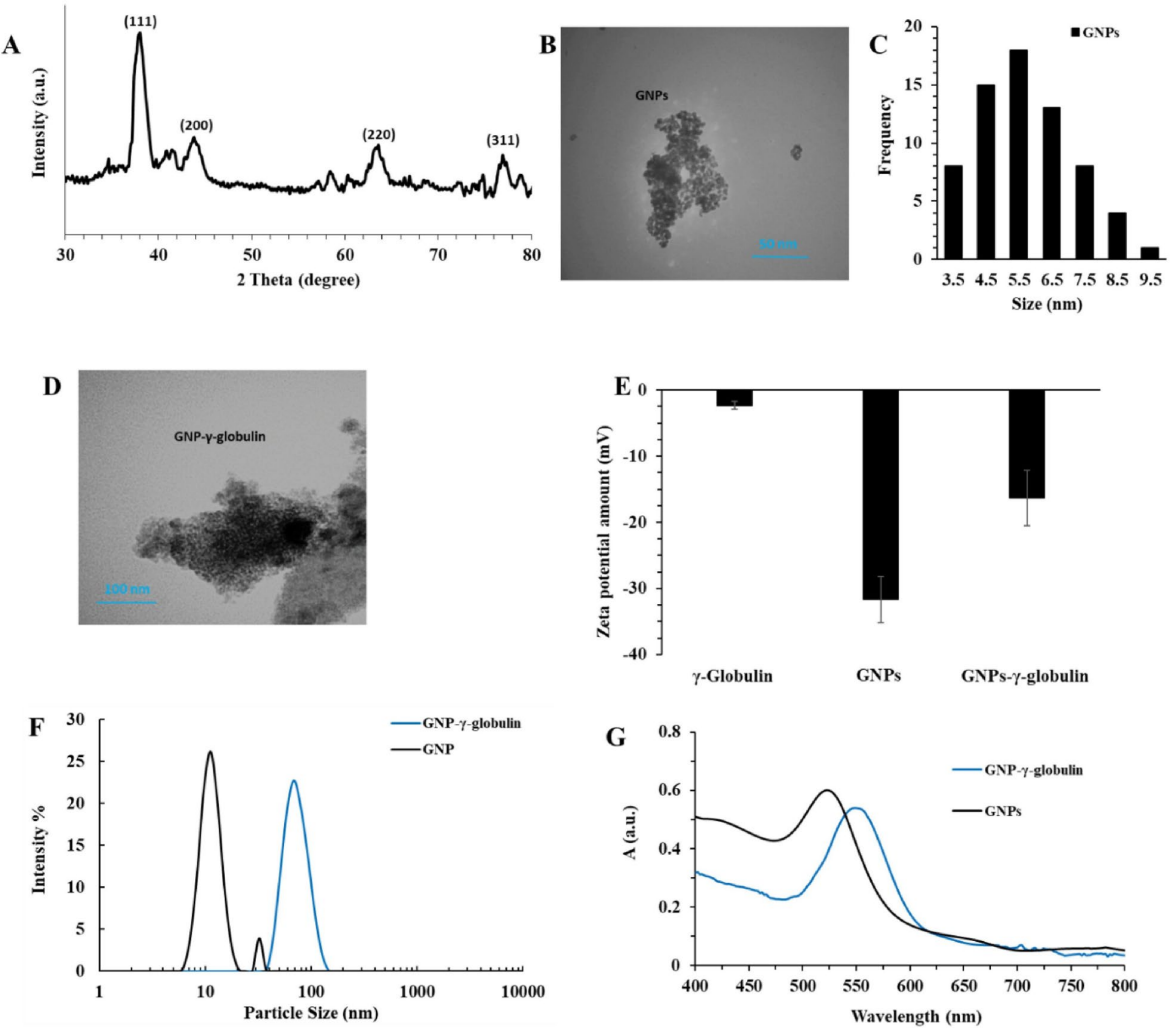
Characterization of ultrasmall GNPs–γ-globulin. **A** Representative XRD pattern of synthesized ultrasmall GNPs. **B** Representative TEM micrograph showing the morphology of (**B**) bare GNPs and (**C**) particle-size distribution of the bare GNPs. **D** TEM micrograph of GNPs–γ-globulin. The corresponding dynamic light scattering (DLS) profiles illustrate the (**E**) the zeta potential and (**F**) hydrodynamic size distribution of bare GNPs and GNPs–γ-globulin, indicating an increase in particle size after interaction with protein. **G** The UV-vis spectra of bare GNPs and GNPs–γ-globulin

XRD is typically employed to analyze the phases and the crystalline nature of NPs Figure 1A. Exhibits the production of face-centered cubic (FCC) GNPs derived from XRD analysis, which is in agreement with the FCC structure reported “in joint committee of powder diffraction standard (JCPDS) card no. 04-0784” [19]. Moreover, the XRD peaks at 2θ of 38.15°, 44.00°, 63.70°, and 77.35° could be associated with the “(111), (200), (220), and (311) crystallographic planes” [19], revealing that the synthesized GNPs crystallize in the cubic system. These data corroborate previous studies on the synthesis of GNPs by other chemical methods [20, 21].

Figure 1B exhibits TEM image of bare GNP. The particle-size distribution of the bare GNPs was determined from the TEM micrograph by measuring the diameters of *N* = 67 individual NPs [22]. The histogram was constructed according to Sturges’ criterion, C = 1 + 3.322 log₁₀(N), resulting in 7 histogram classes (Fig. 1C). The pristine GNPs showed an average particle diameter of 6 ± 2 nm, with a size dispersity of 29.6%. These results confirm the formation of small, nearly spherical GNPs with a relatively homogeneous size distribution. It was detected that although synthesized GNPs in the bare state tend to agglomerate slightly (Fig. 1B), the dispersion level of GNPs in the presence of γ-globulin significantly reduced (Fig. 1D), revealing the aggregation tendency of synthesized GNPs after the interaction with γ-globulin. Indeed, following the addition of γ-globulin to the GNP solution (molar ratio 1:1), the corresponding interaction and the formation of GNPs–γ-globulin complex could mitigate the colloidal stability of GNPs. These data are in good agreement with Mobasherat Jajroud et al. study [23], which reported that the interaction of cerium oxide NPs with hemoglobin can reduce their colloidal stability.

To analyze the aggregation mechanism of GNPs in the presence of γ-globulin, DLS analysis was performed. The particle size distribution and zeta potential of bare GNPs and GNPs–γ-globulin complex were then determined with DLS. This analytical method yields some useful information regarding the hydrodynamic radius and magnitude of repulsion forces between NPs which are the main parameters for determining the colloidal stability of NPs.

Figure 1E revealed that the mean zeta potential values of γ-globulin, GNPs and GNPs–γ-globulin complex were − 2.3 mV ± 0.6, -32 mV ± 4, and − 16 ± 4 mV, respectively. These data indicated that the interaction of GNPs with γ-globulin results in a remarkable decrease in the zeta potential value of GNPs, which may be the main reason for the reduction of the colloidal stability of GNPs [23, 24]. Zeta potential analysis was also supported by mean size calculation determined by DLS. It was revealed that the mean size of bare GNPs was around 11 ± 2 nm while following the addition of γ-globulin to the GNP solution, the mean size of GNPs–γ-globulin was increased to 69 ± 8 nm (Fig. 1F). This observed increase in the mean size of GNPs after the interaction with γ-globulin could be associated with the reduction of zeta potential values and agglomeration of NPs in the presence of this model protein [23]. Indeed, following the interaction of GNPs with γ-globulin and the associated reduction of zeta potential could result in the minimization of the charge-charge repulsion among GNPs [23]. To further verify this claim, UV-visible spectroscopy measurements were done.

UV–visible spectroscopic method is a widely employed strategy for analyzing the formation of NPs by investigating their typical optical characteristics, which are dependent on the physicochemical properties of NPs. The synthesized GNPs are characterized by a significant surface plasmon resonance (SPR) band at 524 nm in the UV–vis spectra (Fig. 1G), revealing the excitation of free electrons following absorbing visible light by NPs [19]. According to *Mie’s theory*, spherical NPs typically show a single SPR band in the absorption spectrum [25]. Also, it was detected that after the interaction of GNPs with γ-globulin, a wider SPR peak was observed at 550 nm, implying that GNPs in the presence of γ-globulin show broader size distributions. In fact, UV–vis spectra of bare GNPs and GNPs–γ-globulin showed that following the addition of γ-globulin the SPR peak is red-shifted from 524 nm for bare GNPs to around 550 nm for complex state, suggesting GNP agglomeration in the presence of protein. Therefore, UV-visible data in line with the DLS data confirmed that GNPs agglomerated apparently in the presence of γ-globulin.

Taken together, solvent-polarity-controlled microwave-assisted synthesis employed in this study enabled the rapid production of ultrasmall GNPs using a simple and energy-efficient protocol. The small particle size obtained through this approach was particularly advantageous for investigating nano–bio interactions because ultrasmall GNPs possess a high surface-area-to-volume ratio and are highly susceptible to surface modification by adsorbed proteins. Consequently, the synthesized GNPs provided an effective model system for elucidating how γ-globulin corona formation influences nanoparticle physicochemical properties and biological responses.

### Effect of GNPs on γ-globulin fluorescence intensity

**Fig. 2:**
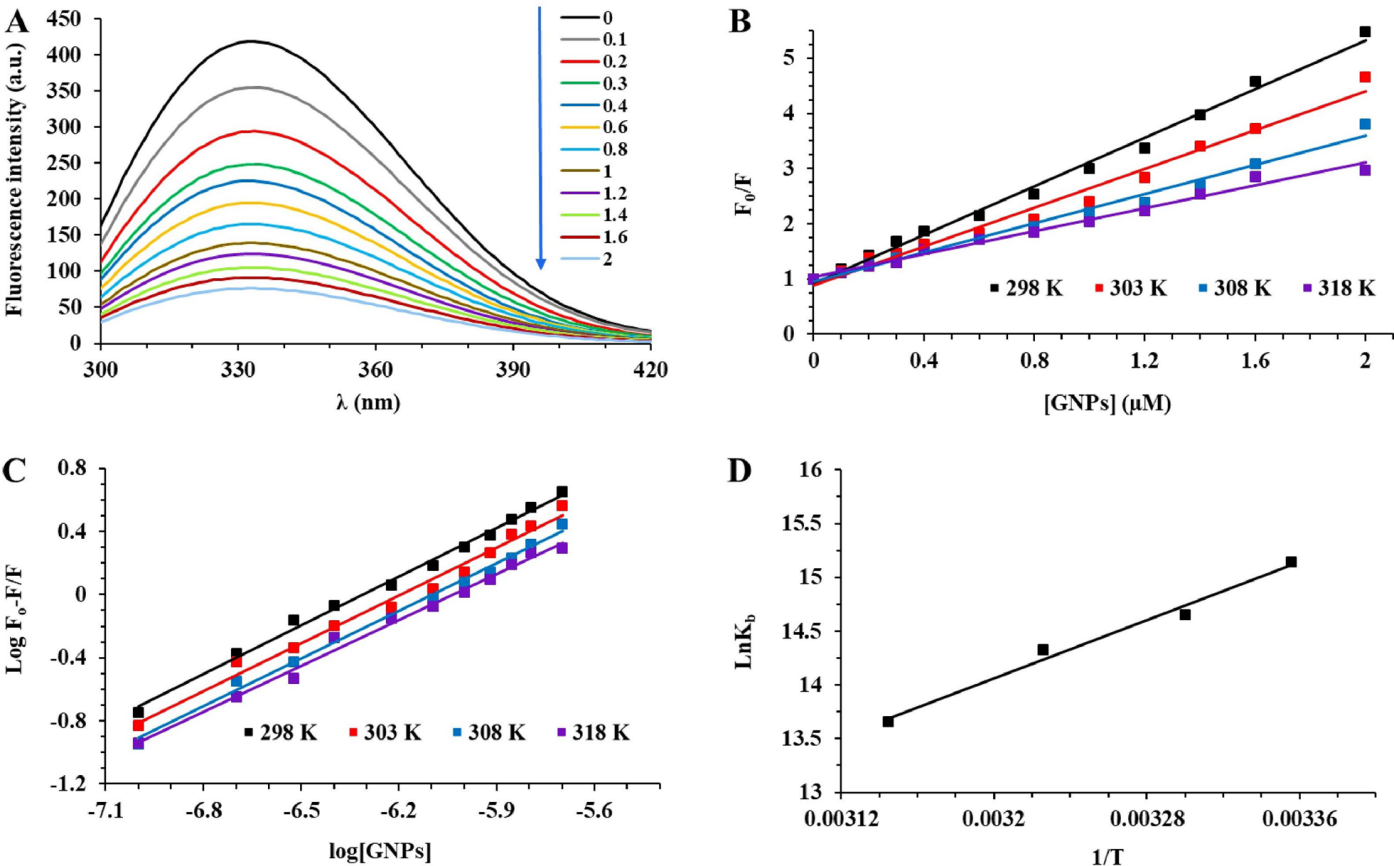
Characterization of ultrasmall GNPs interaction with γ-globulin via fluorescence spectroscopy. **A** Fluorescence intensity measurement for the interaction of γ-globulin and different concentrations of ultrasmall GNPs in the range of 0.1–2 µM. **B** Representative Stern-Volmer plots for the interaction of γ-globulin and ultrasmall GNPs. **C** Representative modified Stern-Volmer plots for the interaction of γ-globulin and ultrasmall GNPs. **D** Representative van’t Hoff plot for the interaction of γ-globulin and ultrasmall GNPs

The influence of GNPs on the emission intensity of γ-globulin at 298 K is shown in Fig. 2A. When the γ-globulin solution is excited at 280 nm, it exhibits a strong emission maximum around 335 nm mainly due to its aromatic residues [26]. GNPs show no apparent emission intensity at the range of studied wavelength, and their contribution to γ-globulin intensity is negligible. However, in the presence of GNPs, the emission intensity of γ-globulin declines in a concentration-dependent fashion.

These data indicate that GNPs can potentially interact with γ-globulin and serve as quenchers to reduce the fluorescence intensity of this protein [13].

#### Fluorescence quenching mechanism

Dynamic quenching or static quenching following the interaction of ligands and proteins derive from the collisions between the quencher ligand and the aromatic residues during the lifetime of the excited state or the formation of a new nonfluorescent complex between the aromatic residues and the ligand. Since we have already detected the intrinsic fluorescence quenching of γ-globulin in the presence of GNPs, to investigate further the quenching mechanism, the emission data were analyzed using the Stern-Volmer (SV) Eq. (2) [27]:

2 $$F_{0}/F=1+k_{q } \tau 0 [Q]=1+KSV[GNPs]$$

where *F*_0_ and *F* show the steady-state emission intensities before and following the incubation of proteins with a quencher, respectively. *K*_SV_ denotes the SV quenching constant, [GNPs] is the ligand concentration, *k*_q_ is the rate constant of bimolecular quenching, and *τ*_0_ is the lifetime of the fluorophore (4.13 ns) [26].

The SV plots of γ-globulin following the interaction with GNPs at four different temperatures were exhibited in Fig. 2B. A good linear relationship of the SV plots reveals that either dynamic or static quenching mechanism (dynamic or static) mediates the formation of GNPs–γ-globulin complex.

Temperature effect is a typical approach to distinguish between dynamic and static quenching mechanisms [26]. For dynamic and static quenching mechanisms, temperature can result in enhancement and reduction of the *K*_SV_ values, respectively. Another potential distinction criterion between dynamic and static quenching mechanisms is the *k*_q_ value. A maximum *k*_q_ value of 2 × 10^10^ L mol^− 1^ *s*^− 1^ reveals that the interaction between ligand and protein mostly arises from dynamic quenching [26]. The values of *K*_SV_ determined from the slope of SV plots (Fig. 2B) were tabulated in Table 1.

**Table 1:** The values of fluorescence quenching of γ-globulin after interaction with ultrasmall GNPs

Gold	Size (nm)	T (K)	K_SV_ (L mol^− 1^)	k_q_ (L mol^− 1^ s ^− 1^)	*R*_2_	Ref.
GNPs	6	298	2.20 × 10^6^	5.08 × 10^14^	0.99	This study
		303	1.75 × 10^6^	4.23 × 10^14^	0.98	
		308	1.32 × 10^6^	3.19 × 10^14^	0.98	
		318	1.04 × 10^6^	2.51 × 10^14^	0.98	
GNPs	10	298	3.009 × 10^8^	3.009 × 10^16^	0.99	[13]

It was manifested that not only the *K*_SV_ values reduce with increasing temperature for GNP–γ-globulin system but also the *k*_q_ values are greater than 2 × 10^10^ L mol^− 1^ *s* ^− 1^, which means that the quenching mechanism of γ-globulin by GNPs is stemmed from static quenching with the formation of a complex [13, 26]. Although the temperature-dependent SV analysis and the exceptionally high *k*_*q*_ values strongly support a static quenching mechanism, fluorescence lifetime measurements were not conducted in the present study. Therefore, future investigations using time-resolved fluorescence spectroscopy would be valuable for directly assessing excited-state lifetime changes and providing additional confirmation of the ground-state complex formation between GNPs and γ-globulin.

#### Binding parameters

The fluorescence intensities for a protein following the interaction with ligands could obey the following Eq. (3) [13]:

3 $$\mathrm{logF}_{0}-\mathrm{F}/\mathrm{F}=\mathrm{logK}_{b}+\mathrm{nlog} [\mathrm{GNPs}]$$

where *F*_0_, *F*, and [GNPs] show the same description as in Eq. (1). *K*_b_ and *n* are the binding constant and the number of binding sites per protein. By the plot of log(*F*_0_-*F*)/*F* vs. log[Q], *K*_b_ *and n* values between GNPs and γ-globulin can be estimated (Fig. 2C; Table 2).

From Fig. 2C, we can observe that the plots show good linearity, indicating that the interaction of GNP and γ-globulin with a single-binding mode agrees well with Eq. (2). The data listed in Table 2 describes that the *K*_b_ values between GNPs and γ-globulin reduce with the increasing of temperature and the binding affinities are all strong with the values with a magnitude of 10^5^–10^6^ L mol^− 1^. This means that temperature rising could lead to a reduction in the stability of the formed system and the interaction between GNPs and γ-globulin is mostly exothermic [13, 26]. The *n* values at all studied temperatures are all about 1, indicating that a single binding site is available in γ-globulin for GNPs.

**Table 2:** The values of binding constants of GNPs–γ-globulin complex

Gold	Size (nm)	T (K)	logK_b_	*n*	*R*_2_	Refs.
GNPs	5	298	6.50	1.03	0.99	This study
		303	6.29	1.01	0.98	
		308	6.15	1.00	0.99	
		318	5.89	0.97	0.99	
GNPs	10	298	≈ 9	1.18	–	[13]
Citrate-coated GNPs	5	298	≈ 5	1.08	0.99	[28]

#### Thermodynamic parameters and binding forces

The values of enthalpy change (ΔH°), entropy change (ΔS°) and free energy change (ΔG°) are closely associated with the binding mechanism. If ΔH° is almost constant, the values of ΔH° and ΔS° can be calculated based on the van’ t Hoff Eq. (4) [26]:

4 $$\mathrm{lnkb}=-\Delta \mathrm{H}/\mathrm{RT}+\Delta \mathrm{S}/\mathrm{R}$$

where *K*_b_ is the binding constant, *T*, is the absolute temperature, and *R* is the gas constant. Then, ΔH° and ΔS° values can be estimated from the slope and intercept of Eq. (3), respectively (Fig. 2D).

The value of ΔG° can be then determined from the Gibbs-Helmholtz Eq. (5) [13]:

5 $$\Delta \mathrm{G}=\Delta \mathrm{H}-\mathrm{T}\Delta \mathrm{S}$$

The thermodynamic values at each studied temperature were summarized in Table 3. The negative ΔG° value reveals the spontaneity of the interaction of GNPs with γ-globulin. The binding of GNP–γ-globulin system was an exothermic process revealed by the negative ΔH° value. The negative ΔS° values also indicated that the interaction between GNPs and γ-globulin is less entropy-driven and the enthalpy is favorable for this reaction [26].

**Table 3:** The values of thermodynamic parameters of GNPs–γ-globulin complex

Gold	Size (nm)	T (K)	ΔH° (kJ mol^− 1^)	ΔS° (J mol^− 1^ K^− 1^)	ΔG° (kJ mol^− 1^)	Ref.
GNPs	5	298	− 56.46	− 63.41	− 37.57	This study
		303			− 37.25	
		308			− 36.93	
		318			− 36.30	
GNPs	10	298	155.302	710.180	− 56.332	[13]

The interaction process between NPs and proteins mainly includes both hydrophilic and hydrophobic forces. The negative ΔH° value can be primarily attributed to hydrophilic interactions; hydrogen bonding and van der Waals interactions are typically associated with relatively large enthalpic contributions [29]. Therefore, ΔH°< 0 as well as ΔS°< 0 between GNPs and γ-globulin as listed in Table 3 signifies that hydrophilic interactions such as hydrogen bonding and van der Waals interaction most likely play a key role in the binding of GNP to γ-globulin. To further analyze the binding forces between GNPs and γ-globulin, a molecular docking study was performed.

### Molecular docking study

The molecular docking study was intended to investigate local Au–protein interactions and adsorption sites, rather than reproduce the entire nanoparticle geometry.

To further analyze the binding properties of GNP surface to γ-globulin structure, molecular docking was applied. Docking simulation for GNP surface–γ-globulin complex could lead to the estimation of energies, forces, and binding sites.

**Fig. 3:**
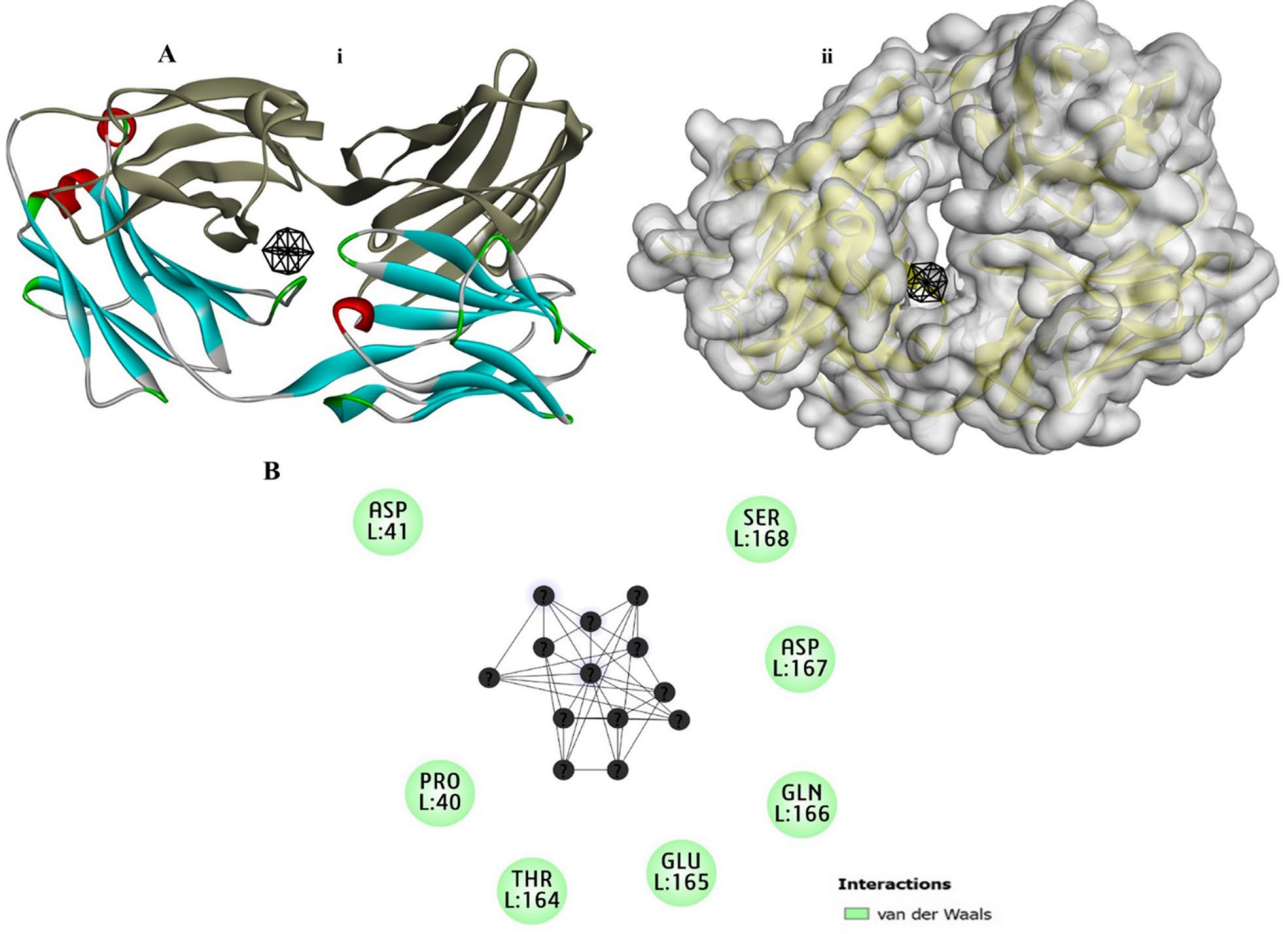
Interaction of ultrasmall Au cluster as a model of GNP surface with a size of 13 atoms with γ-globulin characterized via molecular docking study. **A** Visualization of the interaction of the Au cluster with a size of 13 atoms and γ-globulin in a (i) ribbon model and a (ii) space model. **B** The amino acid residues in the binding pocket of γ-globulin and ultrasmall GNPs

The interaction of the Au cluster as a model of the GNP surface with a size of 13 atoms was visualized in Fig. 3A (i, ii). This conformation was selected with the lowest binding energy of − 9.24 kJ/mol as the best conformer. The Au cluster is positioned in the γ-globulin structure, surrounded by Pro 40, Asp 41, Thr 164, Glu 165, Gln 166, Asp 167, and Ser 168 (Fig. 3B). It was detected that all residues contribute to the formation of van der Waals forces between γ-globulin and GNP surface. This data is confirmed by previous results determined by fluorescence quenching study.

**Fig. 4:**
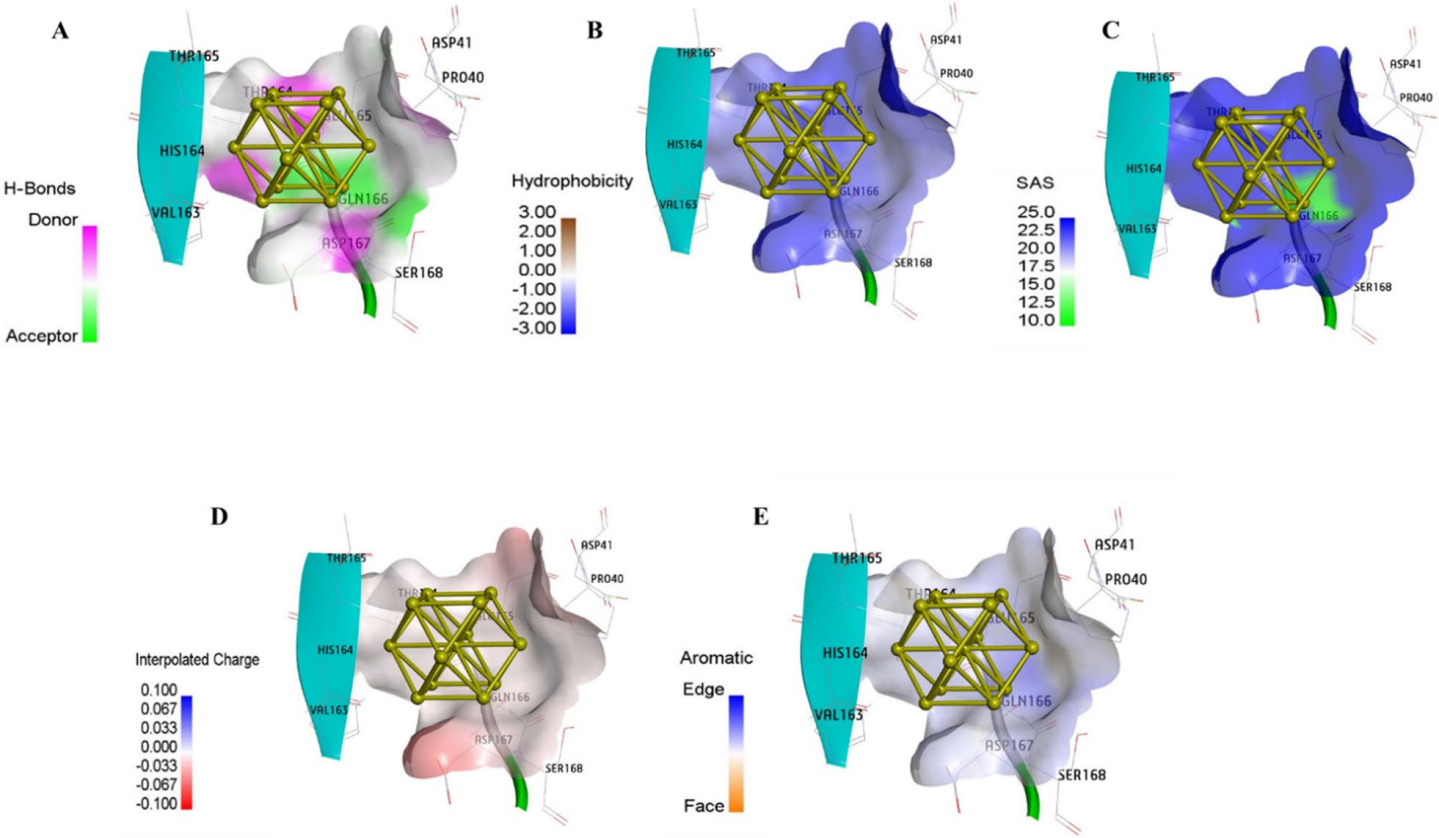
Characterization of the interaction forces after binding of ultrasmall Au cluster as a model of GNP surface with a size of 13 atoms and γ-globulin detected via molecular docking study. Distribution of (**A**) H-bonds, (**B**) Hydrophobicity, (**C**) solvent-accessible surface (SAS), (**D**) interpolate charges, and (**E**) aromatic-mediated forces in the binding pocket of γ-globulin and ultrasmall GNPs

The analyses of H-bonds (Fig. 4A), hydrophobicity (Fig. 4B), solvent-accessible surface (SAS, Fig. 4C), interpolated charge (Fig. 4D) and aromatic residues (Fig. 4E) following the interaction of Au cluster with a size of 13 atoms and γ-globulin by molecular docking study exhibited that both H-bond donor (Thr 164, Asp 167) and acceptor (Gln 166) residues are presented in the binding pocket and hydrophobic interaction are dominant forces in the binding affinity of γ-globulin with Au cluster with a size of 13 atoms. Moreover, charged, and aromatic residues do not play a key role in the interaction of Au cluster with a size of 13 atoms and γ-globulin. Also, SAS analysis revealed that GNP surface interact with the interior part of the protein.

**Fig. 5:**
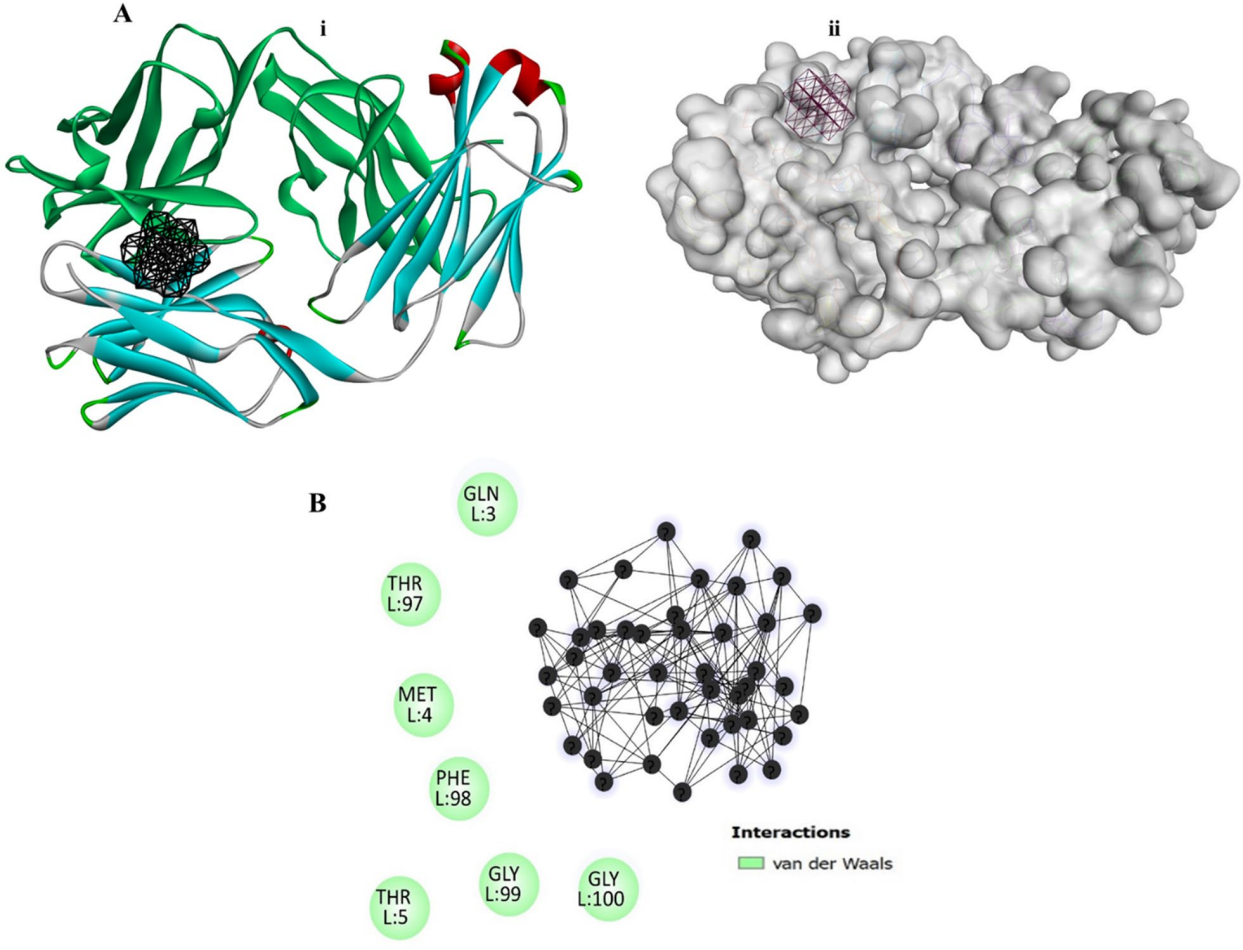
Interaction of ultrasmall Au cluster as a model of GNP surface with a size of 43 atoms with γ-globulin characterized via molecular docking study. **A** Visualization of the interaction of the Au cluster with a size of 43 atoms and γ-globulin in a (i) ribbon model and a (ii) space model. **B** The amino acid residues in the binding pocket of γ-globulin and ultrasmall GNPs

The same procedure was also done to explore the interaction of Au cluster as a model of GNP surface with a size of 43 atoms and γ-globulin. The possible binding site of Au cluster with a size of 43 atoms on γ-globulin structure with the lowest energy conformation (− 12.04 kJ/mol) was shown in Fig. 5A (i, ii). As observed, the amino acid residues contributed to the interaction of Au cluster with a size of 43 atoms and γ-globulin were mainly Gln 3, Met 4, Thr 5, Thr 97, Phe 98, Gly 99, and Gly 100 (Fig. 5B). When compared to Au cluster with a size of 13 atoms, it was seen that both Au clusters interact with γ-globulin by van der Waals forces.

**Fig. 6:**
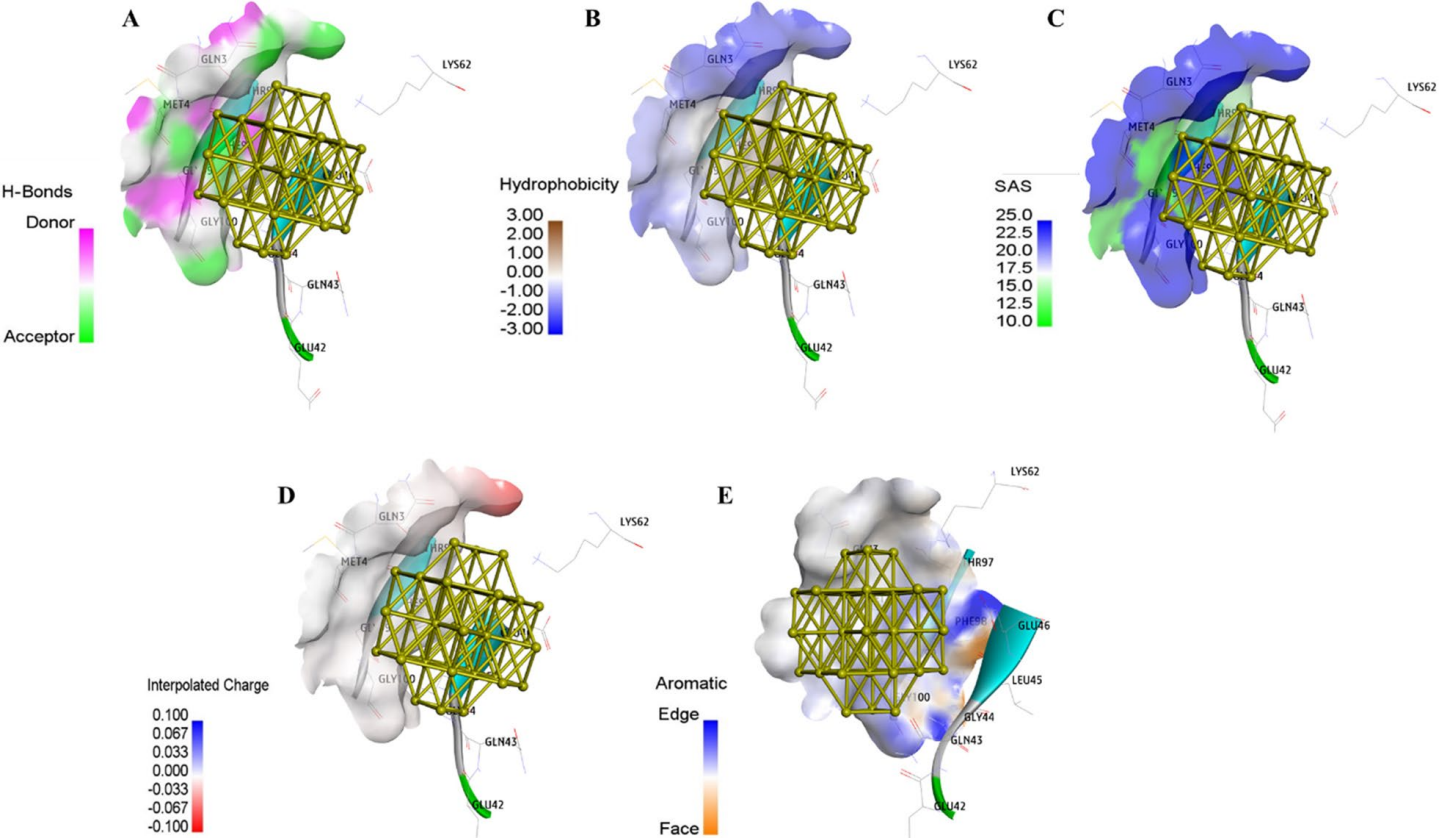
Characterization of the interaction forces after binding of ultrasmall Au cluster as a model of GNP surface with a size of 43 atoms and γ-globulin detected via molecular docking study. Distribution of (**A**) H-bonds, (**B**) Hydrophobicity, (**C**) solvent-accessible surface (SAS), (**D**) interpolate charges, and (**E**) aromatic-mediated forces in the binding pocket of γ-globulin and ultrasmall GNPs

The analyses of H-bonds (Fig. 6A), hydrophobicity (Fig. 6B), SAS, (Fig. 6C), interpolated charge (Fig. 6D) and aromatic residues (Fig. 6E) after the interaction of Au cluster with a size of 43 atoms and γ-globulin indicated that both H-bond donor and acceptor residues are presented in the binding pocket and hydrophobic, charged, and aromatic residues do not play a key role in the interaction of Au cluster with a size of 43 atoms and γ-globulin. Also, SAS analysis revealed that GNP surface mostly interact with the surface of protein and attach to the exterior part of the protein.

A negligible number of hydrophobic and interpolated charge forces were formed in the interaction between designed Au clusters and γ-globulin, which is consistent with the outcomes extracted from the thermodynamic parameters.

The molecular structure of γ-globulin plays a crucial role in its interaction with GNPs. γ-Globulin is a β-sheet-rich protein containing numerous amino acid residues with hydroxyl (-OH), amino (-NH_2_), amide (-CONH-), and carboxyl (-COOH) functional groups that can participate in intermolecular interactions with GNP surfaces. The thermodynamic parameters obtained in the present study suggest that hydrogen bonding and van der Waals interactions are the dominant driving forces governing the formation of the GNP–γ-globulin complex. Molecular docking further supported this interpretation by identifying polar residues within the binding regions of the Au clusters. These residues provide hydrogen-bond donor and acceptor sites that facilitate adsorption onto the GNP surface. The resulting protein corona may modify the surface characteristics of GNPs, leading to changes in colloidal stability, cellular uptake, and biological activity.

Taken together, the docking analyses demonstrate that increasing cluster size from Au13 to Au43 alters the accessible binding regions on the γ-globulin surface and changes the identity of the interacting amino acid residues. Nevertheless, both clusters interact predominantly through hydrogen bonding and van der Waals interactions, in agreement with the thermodynamic parameters obtained from fluorescence spectroscopy. Therefore, cluster size appears to modulate the geometry and residue composition of the binding interface, while the underlying physicochemical driving forces responsible for complex formation remain essentially unchanged.

Furthermore, the molecular docking and thermodynamic results provide a mechanistic explanation for the physicochemical changes observed following γ-globulin adsorption onto the GNP surface. Because the synthesized GNPs are initially stabilized by negatively charged citrate molecules, adsorption of γ-globulin onto the NP surface can partially shield or neutralize these surface charges through protein–nanoparticle interactions. This phenomenon is consistent with the observed decrease in zeta potential after protein corona formation. The reduction in electrostatic repulsion among neighboring NPs promotes particle association and aggregation, which is reflected by the increase in hydrodynamic diameter. Furthermore, NP aggregation modifies plasmonic coupling between adjacent particles, resulting in the broadening and red-shift of the SPR band. These findings collectively demonstrate how molecular-level interactions between γ-globulin and GNPs directly govern the colloidal stability and optical properties of the resulting nanocomposite.

### Investigation of γ-globulin conformational changes

**Fig. 7:**
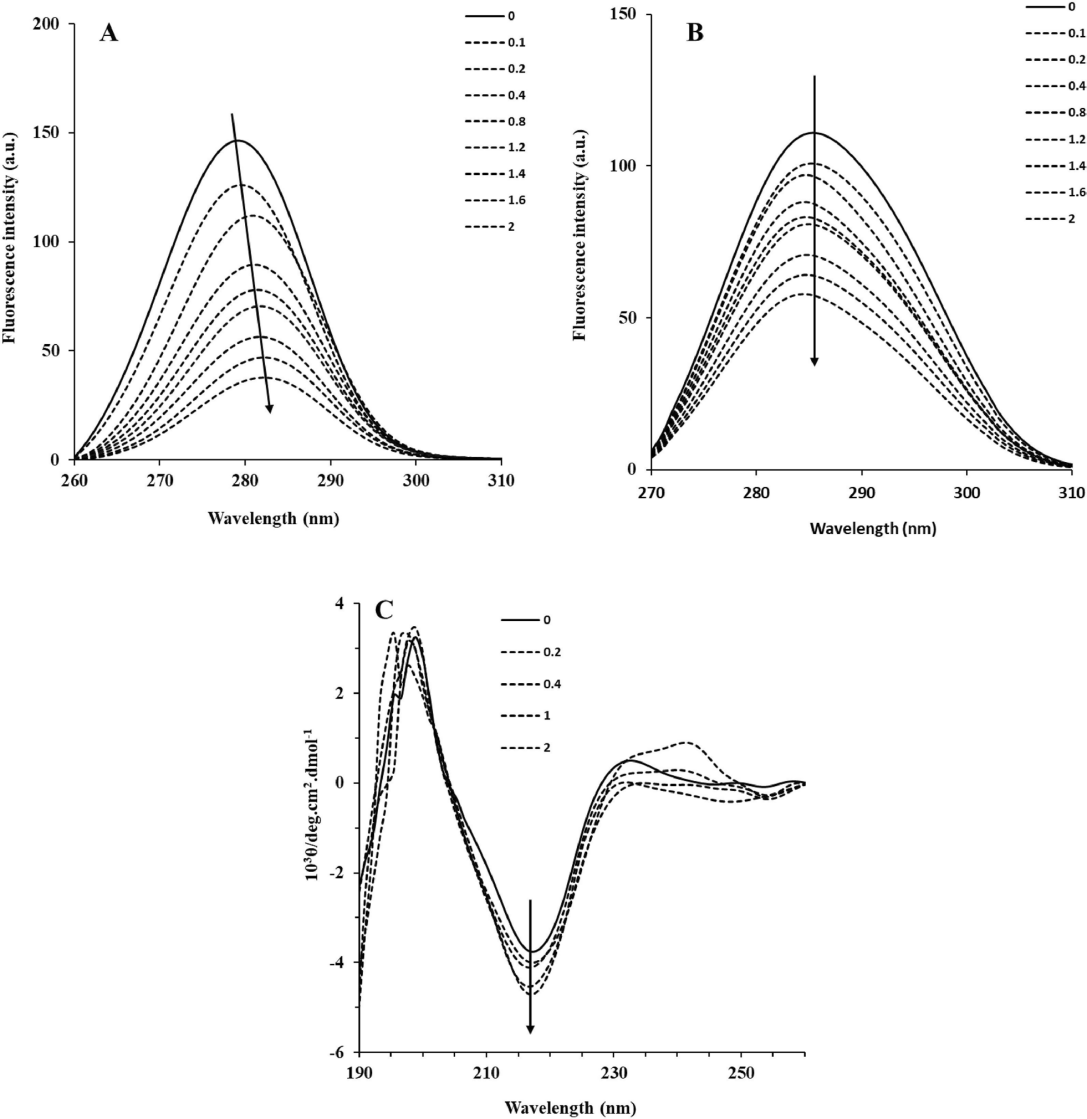
Characterization of γ-globulin structural changes after interaction with ultrasmall GNPs via fluorescence and circular dichroism (CD) spectroscopy methods

Synchronous fluorescence spectra can provide details regarding the molecular microenvironment of the Trp and Tyr residues. Upon fixation of wavelength interval (Δλ) between excitation and emission wavelength at 60 and 15 nm, synchronous fluorescence shows characteristic information of Trp and Tyr residues, respectively. The fluorescence quenching along with blue or red shift of the λ_max_ implicates the microenvironmental changes around Tyr or Trp residues [30]. The synchronous fluorescence spectra of γ-globulin following the interaction with GNPs were shown in Fig. 7A (Δλ = 60 nm) and Fig. 7B (Δλ = 15 nm). The fluorescence quenching of Trp residue had a red shift upon the addition of GNPs (Fig. 7A), whereas only moderate quenching and shift were observed when the experiment was done at Δλ = 15 nm (Fig. 7B). The data implies that GNPs might interact with protein in the vicinity of Trp residues and increase the polarity surrounding these residues but show no significant effect on the microenvironmental changes around Tyr-residues.

Interestingly, molecular docking analysis did not identify aromatic residues as direct constituents of the primary binding pockets for either the Au13 or Au43 clusters. This finding is not inconsistent with the synchronous fluorescence results. Synchronous fluorescence spectroscopy is highly sensitive to subtle alterations in the local microenvironment surrounding aromatic fluorophores and, therefore, does not necessarily imply direct GNP–residue contact. Consequently, the red shift observed for Trp residues is more likely attributable to localized conformational rearrangements of γ-globulin and/or changes in the solvent accessibility and polarity of the Trp microenvironment induced by GNP binding at adjacent regions of the protein. These observations suggest that the interaction of GNPs with γ-globulin indirectly perturbs the structural environment of aromatic residues without requiring their direct participation in the nanoparticle-binding interface.

The synchronous fluorescence spectra of γ-globulin following the interaction with different concentrations of ultrasmall GNPs in the range of 0.1 to 2 µM at (A) Δλ = 60 nm and (B) Δλ = 15 nm. (C) The far-UV CD spectra of γ-globulin following the interaction with different concentrations of ultrasmall GNPs in the range of 0.2 to 2 µM.

Circular dichroism (CD) is mostly used to detect conformational changes of proteins following the interaction with NPs due to its accuracy and sensitivity. Far-UV CD spectra (190–240 nm) is frequently used to determine the secondary structure of a protein [31]. The characteristic CD peaks can reflect the changes in the content of the secondary structure of proteins, α-helix, β-sheet, and random coil. To gather some information regarding the secondary structural changes of γ-globulin following the interaction with GNPs, far-UV CD spectra (190–260 nm) were performed. The CD spectra of free γ-globulin and GNPs–γ-globulin system were exhibited in Fig. 7C. The CD spectrum of native protein shows one negative band in the ultraviolet region around 217 nm, which is an associated model of the β-sheet structure [26]. In Fig. 7C, although the chirality of γ-globulin partially increases following the interaction of the protein with different concentrations of GNPs, no significant changes in the shape and position of CD spectra at 217 nm were observed. This data signify that GNPs did not lead to a substantial alteration in the β-sheet content of γ-globulin. The detected data are consistent with the previous reports [28, 13], which revealed that upon the interaction of GNPs (10 nm) and citrate-coated GNPs (5 nm), respectively, the secondary structure of γ-globulin remained almost intact.

The results pointed out slight conformational changes in the secondary structure of γ-globulin upon the interaction with ultrasmall GNPs.

### Anticancer and biocompatibility evaluation of GNPs and GNPs–γ-globulin complex

**Fig. 8:**
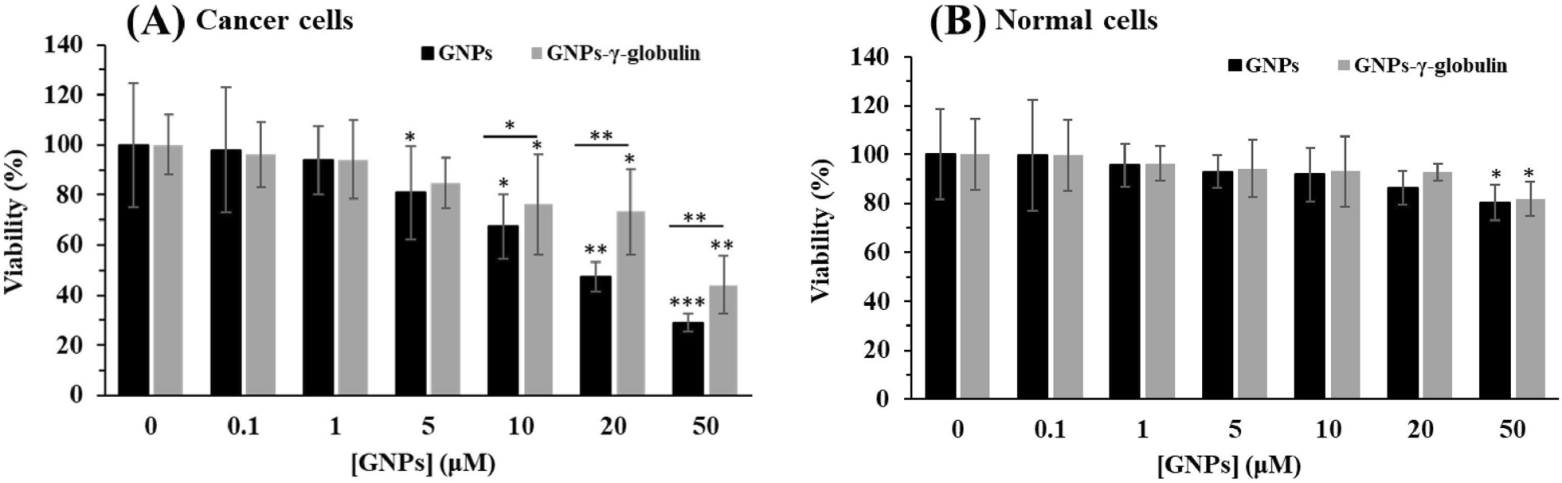
The influence of the γ-globulin protein corona on GNP-induced growth inhibition. MTT assay for the evaluating the anti-proliferative effects of ultrasmall GNPs and GNP–γ-globulin complex on (**A**) HCC HepG2 and (**B**) normal human hepatic L-02 cells after 24 h of treatment. The results demonstrate *P < 0.05, **P < 0.01, ***P < 0.001 versus control

The MTT assay was done to evaluate the anti-growth effect and biocompatibility of GNPs and GNPs–γ-globulin complex on HCC HepG2 and normal human hepatic L-02 cells. The data showed that although GNPs significantly mitigated the growth in HCC HepG2 cells, GNPs–γ-globulin complex showed a less remarkable effect towards the growth inhibition of the cells (Fig. 8a). The IC_50_ concentrations of GNPs in the bare state and in the form of complex with γ-globulin in HCC HepG2 cells after 24 h were shown to be 16 ± 2 µM and 250 ± 32 µM, respectively.

The cytotoxic effects of bare GNPs and GNPs–γ-globulin complex on normal human hepatic L-02 cells were evaluated using the MTT assay (Fig. 8b). Cell viability remained relatively high across all tested concentrations, indicating low cytotoxicity of both formulations.

Notably, GNPs–γ-globulin exhibited slightly improved biocompatibility compared to bare GNPs at intermediate concentrations (5–20 µg/mL), where cell viability exceeded 92%. Overall, both NP formulations demonstrated dose-dependent reductions in cell viability; however, the viability remained above 80% even at the highest concentration tested, suggesting acceptable biocompatibility toward healthy cells.

The greater sensitivity of HepG2 cells to GNP treatment compared with normal hepatic L-02 cells may be attributed to fundamental variations in cellular metabolism and NP uptake behavior. Cancer cells typically display enhanced endocytic activity relative to normal cells, which may facilitate greater GNP internalization and intracellular accumulation.

These data indicated that the presence of γ-globulin on the surface of GNPs, the formation of the protein corona, decreases the proliferation inhibition of HCC HepG2 cells induced by ultrasmall GNPs. This phenomenon is likely due to reduced interactions between GNPs–γ-globulin complexes and the cancer cell membrane, caused by GNP aggregation in the complex form. This data is not in agreement with the results provided by Azizi et al. [32], showing that coated silver NPs by a blood protein such as albumin can increase the anticancer effects of NP-protein complex. The main reason for these different outcomes may be due to the increase of the mean diameter of ultrasmall GNPs and reduction in their zeta potential value upon the interaction with γ-globulin which could result in the NP aggregation and inferior interaction potential with cell membrane accordingly [32]. Also, based on UV-visible study, we found that the SPR intensity of GNPs is reduced upon the interaction with γ-globulin, which can be another reason for the reduction of the anticancer effect of these NPs following the interaction with γ-globulin. In agreement with Yin et al. [33] and Žūkienė et al. [34] we found that varying the bare state of NPs by coating with proteins could result in the reduction of cytotoxicity stimulated by NPs. Therefore, this data highlighted the need to explore thoroughly the influences of NP surface modification by proteins on cytotoxicity assays.

### Intracellular Au content measurements

Intracellular Au contents of HCC HepG2 cells were assessed using ICP optical emission spectroscopy after exposure to 16 µM GNPs for 24 h. It was detected that cellular uptake levels of bare GNPs and GNPs–γ-globulin were 3.87 pg and 1.19 pg, respectively, indicating that the interaction of GNPs with γ-globulin can reduce their cellular uptake. The cellular uptake of NPs can be modulated by engineering their protein corona. GNPs usually interact with cell membranes and trigger membrane wrapping and endocytotic uptake [35]. Based on the data presented here, we conclude that the presence of a protein corona on the surface of GNPs reduces their interaction with cells, thereby decreasing membrane wrapping and subsequent endocytic uptake.

### Induced oxidative stress evaluation of GNPs and GNPs–γ-globulin complex

**Fig. 9:**
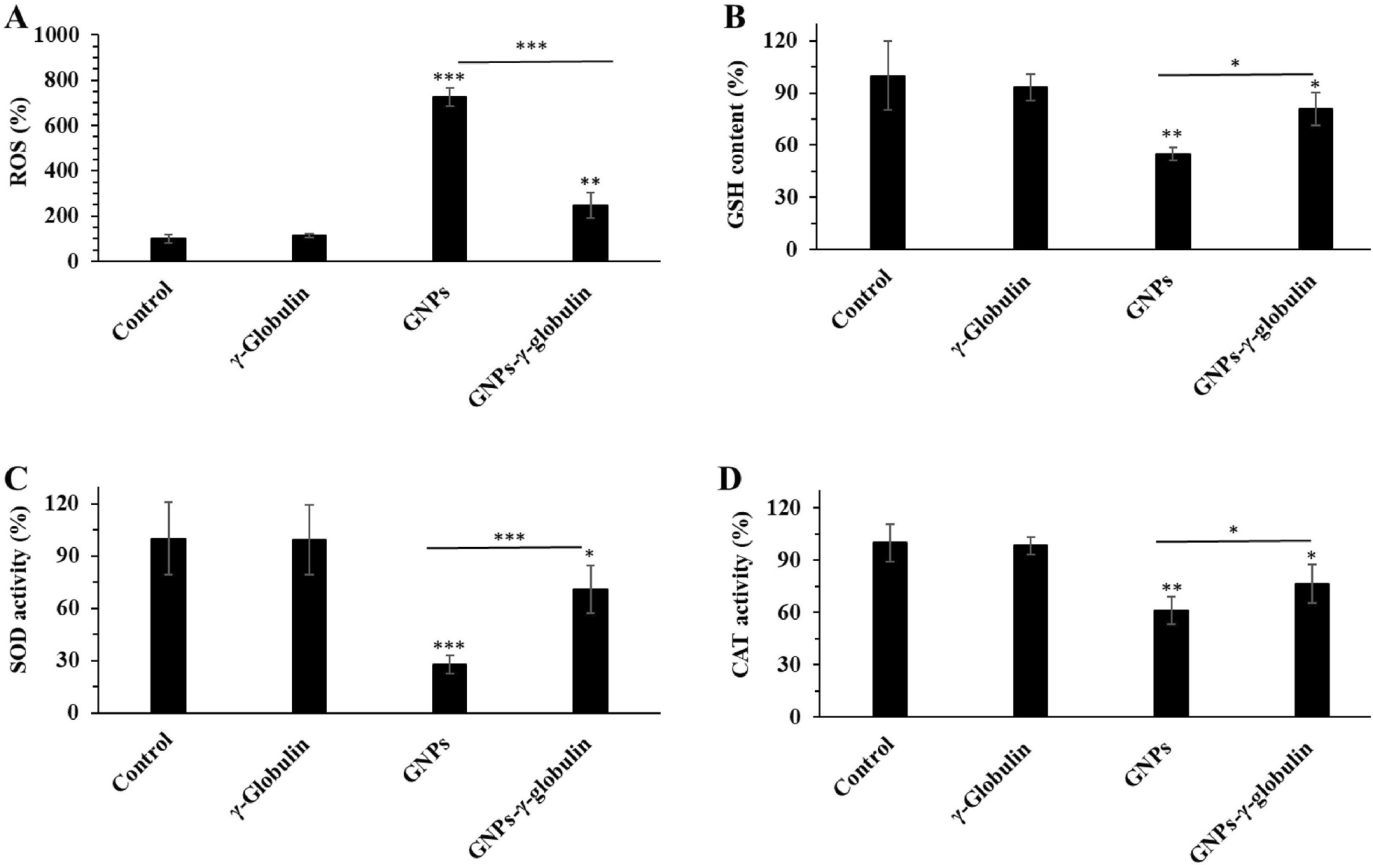
The modulatory effect of the protein corona (γ-globulin coating) on GNP-induced oxidative stress and cytotoxicity. (**A**) ROS levels, (**B**) GSH content, (**C**) SOD activity, and (**D**) CAT activity in HCC HepG2 cells after 24 h exposure to ultrasmall GNPs (16 µM) and the GNP–γ-globulin complex. The results demonstrate *P < 0.05, **P < 0.01, ***P < 0.001 versus control

To investigate the effect of bare GNPs and GNPs–γ-globulin complex on oxidative stress in HCC HepG2 cells, we measured intracellular ROS levels, GSH content, SOD activity, and CAT activity after 24 h of incubation. The excessive production of ROS might cause the disruption of homeostasis balance in the enzymatic and non-enzymatic system of ROS scavenging antioxidants. As shown in Fig. 9A, ROS levels in cells exposed to 16 µM bare GNPs were significantly higher than those in cells treated with GNP–γ-globulin complex. These data suggest that cell death induced by GNPs is regulated by ROS production, which may change the cellular redox status. To further examine this hypothesis, we evaluated GSH content, SOD activity, and CAT activity. The results showed that bare GNPs induced greater reductions in GSH content (Fig. 9B), SOD activity (Fig. 9C), and CAT activity (Fig. 9D) compared with GNP–γ-globulin complex.

We conclude that GNPs induce oxidative stress by disrupting redox homeostasis through the inhibition of both enzymatic and non-enzymatic antioxidant defense systems. In contrast, the interaction of GNPs with γ-globulin reduces their oxidative potential, which is consistent with the observed MTT assay results. Similar findings have been reported by Yin et al. [33] and Žūkienė et al. [34], demonstrating that coating the NP surface with proteins or other agents can reduce cytotoxicity by decreasing ROS production compared with bare GNPs.

### Induced apoptotic evaluation of ultrasmall GNPs and GNPs–γ-globulin complex

Based on the previous section, we can claim that increased ROS levels might result in apoptosis. To further investigate the effects of bare ultrasmall GNPs and GNPs–γ-globulin complex on in HCC HepG2 cells, the apoptotic markers were evaluated.

**Fig. 10:**
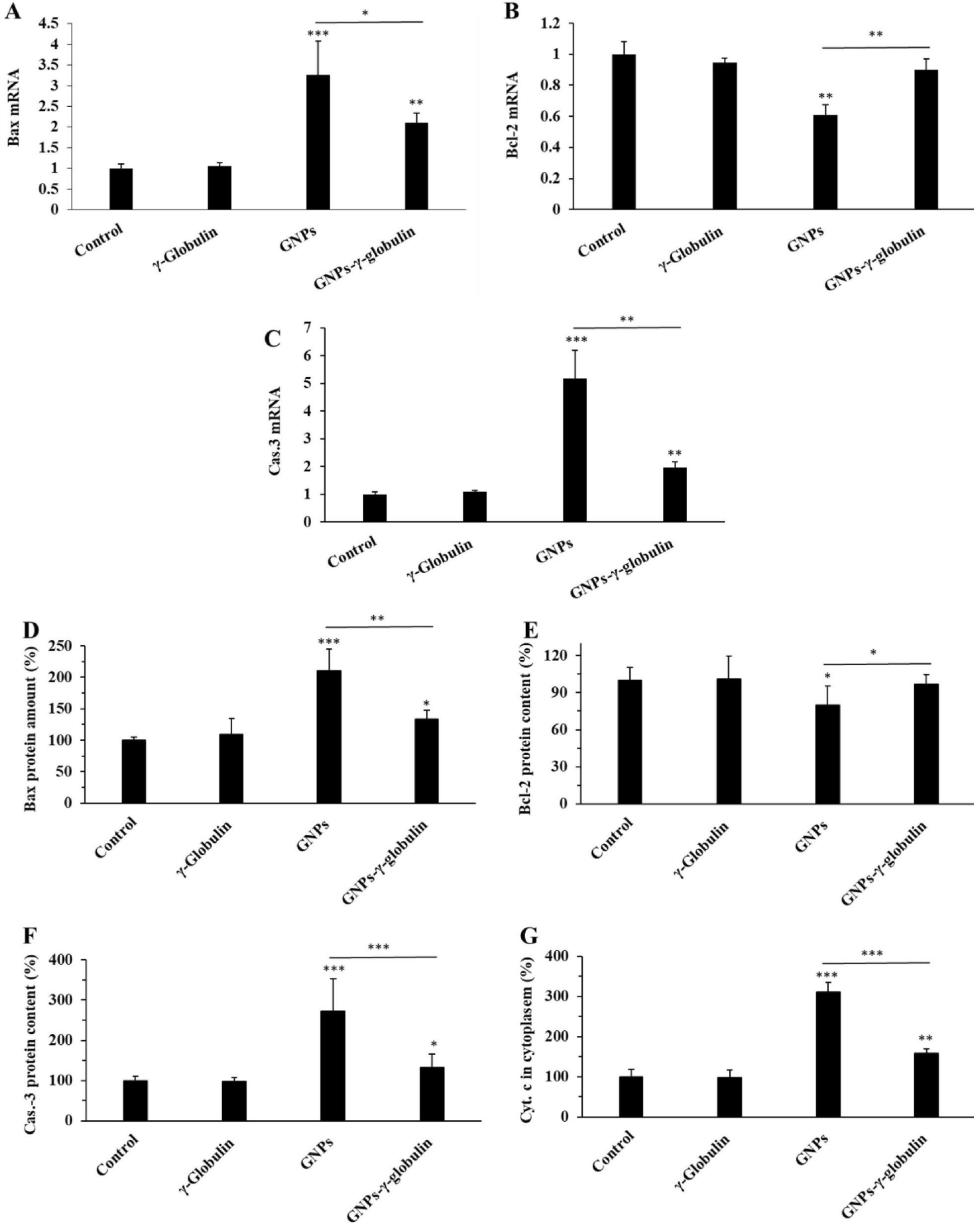
The modulatory effect of the protein corona (γ-globulin coating) on GNP-induced apoptosis. (**A**) Bax mRNA, (**B**) Bcl-2 mRNA, (**C**) Cas.3 mRNA, (**D**) Bax protein, (**E**) Bcl-2 protein, (**F**) Cas.3 protein, (**G**) Cyt.c release assays in HCC HepG2 cells after 24 h exposure to ultrasmall GNPs (16 µM) and the GNP–γ-globulin complex. The results demonstrate *P < 0.05, **P < 0.01, ***P < 0.001 versus control

The main markers of apoptosis are the upregulation of the apoptotic marker, Bax, cytochrome c release, caspase-3, and downexpression of the antiapoptotic marker, Bcl-2. Therefore, we assessed the expression of these markers in the mRNA and protein levels by qRT-PCR and ELISA assays, respectively. As shown in Figs. 10A–C, both bare ultrasmall GNPs (16 µM) and the GNP–γ-globulin complex induced apoptosis in HCC HepG2 cells after 24 h by upregulating Bax and caspase-3 mRNA expression and downregulating Bcl-2 mRNA expression. However, the apoptotic response induced by bare GNPs at 16 µM was significantly greater than that observed for the GNP–γ-globulin complex at the same concentration. Consistent results were also observed at the protein level, as demonstrated by changes in Bax, Bcl-2, and caspase-3 expression detected using ELISA assays (Figs. 10D–F).

The results of cytochrome c release to cytoplasm in HCC HepG2 cells treated with bare ultrasmall GNPs and GNPs–γ-globulin complex shown in Fig. 10G, also indicated that both GNP states induced the cytochrome c release in cancer cells, however, there was a significant difference between bare GNPs and GNPs–γ-globulin complex impact on upregulation of this marker. Previous studies by Zhang et al. [36] demonstrated that the cancer cell line treated with biosynthesized GNPs with a size of around 100 nm exhibited the same apoptotic pattern as we detected. However, Yang et al. showed that conformational changes in the albumin after the interaction with ultrasmall GNPs (2.8 nm) could promote the cell-membrane penetration and induced apoptotic effects of GNP- albumin complex [37]. The different results between these studies may be derived from the different physicochemical properties of NPs, different types of proteins and cells used for these reports and induced conformational changes of protein [38, 39]. In agreement with our results, Nayak et al. reported that lactoferrin interaction with silver NP interface can reduce the cytotoxicity of these NPs which was due to the minimal structural changes of the adsorbed protein [40]. Also, Jafari et al. reported that human plasma protein (mostly fibrinogen) corona could lead to a significant reduction in the toxicity of metal-organic framework against cancer cells [41].

## Conclusions

This study presents an integrated investigation of the synthesis, nano–bio interactions, and biological activity of ultrasmall GNPs. The GNPs were successfully synthesized through a solvent-polarity-controlled microwave-assisted approach and subsequently employed as a model system to investigate the influence of γ-globulin corona formation on NP interaction. Comprehensive physicochemical characterization demonstrated that adsorption of γ-globulin significantly altered the colloidal properties of GNPs, resulting in changes in hydrodynamic size, zeta potential, and surface plasmon resonance characteristics. Spectroscopic and molecular docking consistently revealed that the interaction between GNPs and γ-globulin is governed primarily by hydrogen bonding and van der Waals interactions, leading to the formation of a stable protein corona with only minor conformational perturbations of the protein structure. Importantly, the study demonstrates that protein corona formation substantially modifies the biological identity of ultrasmall GNPs. Specifically, γ-globulin adsorption reduced GNP cellular uptake, attenuated ROS generation, preserved intracellular antioxidant defenses, and weakened apoptosis-related signaling pathways in HepG2 cells, thereby significantly decreasing the anticancer activity observed for bare GNPs. Unlike conventional studies that primarily focus on the cytotoxic effects of GNPS, the present work establishes a direct mechanistic relationship between protein corona formation, physicochemical transformations, and downstream biological responses. These findings highlight the critical role of plasma proteins in determining NP fate and functionality and underscore the importance of considering nano–bio interface interactions during the rational design of GNP-based therapeutic and drug-delivery platforms.

## Data Availability

The datasets used and/or analyzed during the current study are available from the corresponding author on reasonable request.
